# The role of polyamines in protein-dependent hypoxic tolerance of *Drosophila*

**DOI:** 10.1186/1472-6793-8-22

**Published:** 2008-12-02

**Authors:** Paul Vigne, Christian Frelin

**Affiliations:** 1 Inserm, U615, Nice, F-06108, France; 2 Univ Nice Sophia Antipolis, Nice, F-06108, France

## Abstract

**Background:**

Chronic hypoxia is a major component of ischemic diseases such as stroke or myocardial infarction. *Drosophila *is more tolerant to hypoxia than most mammalian species. It is considered as a useful model organism to identify new mechanisms of hypoxic tolerance. The hypoxic tolerance of flies has previously been reported to be enhanced by low protein diets. This study analyses the mechanisms involved.

**Results:**

Feeding adult *Drosophila *on a yeast diet dramatically reduced their longevities under chronic hypoxic conditions (5% O_2_). Mean and maximum longevities became close to the values observed for starving flies. The action of dietary yeast was mimicked by a whole casein hydrolysate and by anyone of the 20 natural amino acids that compose proteins. It was mimicked by amino acid intermediates of the urea cycle such as L-citrulline and L-ornithine, and by polyamines (putrescine, spermidine and spermine). α-difluoromethylornithine, a specific inhibitor of ornithine decarboxylase, partially protected hypoxic flies from amino acid toxicity but not from polyamine toxicity. N^1^-guanyl-1,7 diaminoheptane, a specific inhibitor of eIF5A hypusination, partially relieved the toxicities of both amino acids and polyamines.

**Conclusion:**

Dietary amino acids reduced the longevity of chronically hypoxic flies fed on a sucrose diet. Pharmacological evidence suggests that the synthesis of polyamines and the hypusination of eIF5A contributed to the life-shortening effect of dietary amino acids.

## Background

Amino acid sensing is now recognized as an essential property of eukaryotic cells. It allows cells from developing organisms to adjust their rate of protein synthesis to the availability of amino acids. The availability/deprivation of amino acids is sensed by specific cellular protein kinases which target essential factors of the protein synthesis machinery. The availability of amino acids is sensed by the TOR pathway [1]. TOR activity is stimulated under nutrient rich conditions, hence leading to the phosphorylation of 4E-BP and an increase in the pool of free, active, eIF4E. GCN2 kinase senses the opposite situation, i.e. the deficiency of a single amino acid species. GCN2 kinase is activated by uncharged tRNAs and it triggers a repression of global protein synthesis by phosphorylating the translation initiation factor eIF2α [2].

Non growing tissues also sense available nutrients. For instance, it is well known that moderate dietary restrictions increase the longevity of a variety of organisms from yeast to mammals [3]. In *Drosophila*, the beneficial action of dietary restriction is largely reproduced by reducing dietary proteins [4-6]. The mechanism involved is not known.

Chronic hypoxia is the situation in which tissues are exposed for long periods of time to oxygen tensions that are less than those required for optimum functioning. Chronic hypoxia is a major consequence of ischemic diseases such as stroke, myocardial infarction and venous thromboembolism and it clearly contributes to the progression of the diseases. The mechanisms by which chronic hypoxia induces hypoxic tissue damage are largely unknown and there is a need for innovative pharmacological strategies to enhance the hypoxic tolerance of ischemic tissues. *Drosophila *is increasingly used as a model system to analyze how whole organisms respond to exogenous stresses such as reduced oxygen [7]. We previously reported that chronically hypoxic flies retain normal activities but age at a faster rate than normoxic flies. We further observed that restriction of dietary proteins dramatically increases the longevity of hypoxic flies [6,8]. In this study we analyse the mechanisms involved in the diet dependent hypoxic tolerance. The results indicate that chronic hypoxic conditions unmask a life shortening effect of dietary amino acids which is mimicked by polyamines and partially prevented by inhibitors of polyamine synthesis. This study thus identifies a new amino acid sensing mechanism which determines the tolerance to chronic hypoxic stresses.

## Results

### Dietary protein and amino acid induced hypoxic death

Laboratory populations of *Drosophila *are usually raised on nutrient mixtures that consist of sucrose as a source of carbohydrates and heat inactivated yeast as a source of proteins and cofactors. We previously assessed the influences of sucrose and yeast on the longevity of flies maintained at atmospheric oxygen tension (21% O_2_, normoxia) or under chronic hypoxic conditions (5% O_2_) [6]. Results indicated that yeast and sucrose increased the longevity of normoxic flies. Sucrose increased the longevity of chronically hypoxic flies. Yeast did not. In addition dietary yeast suppressed the sucrose dependent longevity of chronically hypoxic flies. This paper deals with the mechanisms by which dietary yeast decreased the longevity of chronically hypoxic flies fed on a sucrose diet.

**Figure 1:**
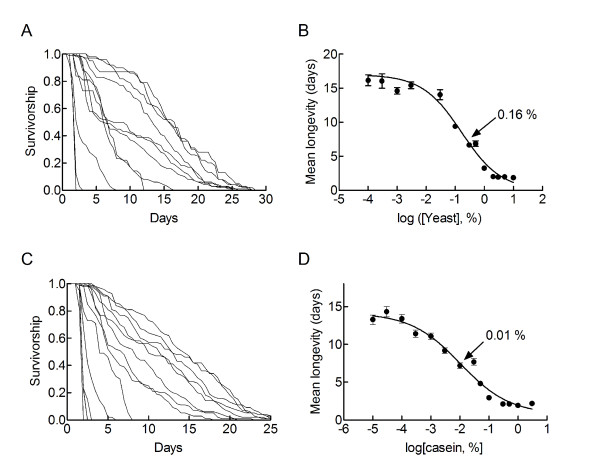
**Heat inactivated yeast and casein reduce the longevity of chronically hypoxic flies**. Flies were exposed to a 10% sucrose diet supplemented with different concentrations of yeast (A, B) or of casein (C, D) and survivorship curves were determined under chronic hypoxia. A, C: Survivorship curves. Note that yeast and casein decreased both the median and maximum longevities. B, D. Dose response curves for the inhibitory action of yeast (B) and casein (D). Mean longevities ± sem are indicated. The half maximum action of yeast was observed at a relative concentration of 0.16% (95% confidence interval: 0.07–0.38%). The half maximum action of casein was observed at 0.01% casein (95% confidence interval: 0.003–0.03%). The total numbers of flies under experimentation were 1722 and 1526 in experiments using yeast and casein respectively. Sample sizes used to define mean longevities were 40–323.

Figure 1A shows survivorship curves of hypoxic flies fed on a 10% sucrose diet supplemented with different concentrations of heat inactivated yeast. Dietary yeast decreased both the median and maximum longevities. Figure 1B presents the dose response curve for the inhibitory action of yeast on the mean survival. It indicated a dose dependent and saturable action of yeast. Half maximum inhibition was observed at 0.16% yeast. Maximally effective concentrations of yeast decreased the mean survival of hypoxic flies to about 2 days. A similar value (2.4 ± 0.1 days, n = 328) was observed for starving flies that were exposed to the same hypoxic conditions.

Heat inactivated yeast is a complex mixture which comprises proteins, carbohydrates, nucleic acids, lipids and a variety of cell metabolites. We used a casein hydrolysate to evaluate the possible contribution of amino acids to the life shortening effect of yeast. Survivorship curves were established for hypoxic flies fed on a 10% sucrose diet supplemented with different concentrations of casein. Figure 1C shows that casein decreased both the median and maximum longevities of hypoxic flies. Figure 1D shows that the action of casein on the mean survival of hypoxic flies was dose dependent and saturable. Maximally effective doses of casein reduced the longevity of hypoxic flies to the same extent as maximally effective concentrations of yeast. The half maximum inhibition was observed at 0.01% casein. Thus, casein had the same maximum efficacy as yeast but it was 16 times more potent on a weight basis.

**Figure 2:**
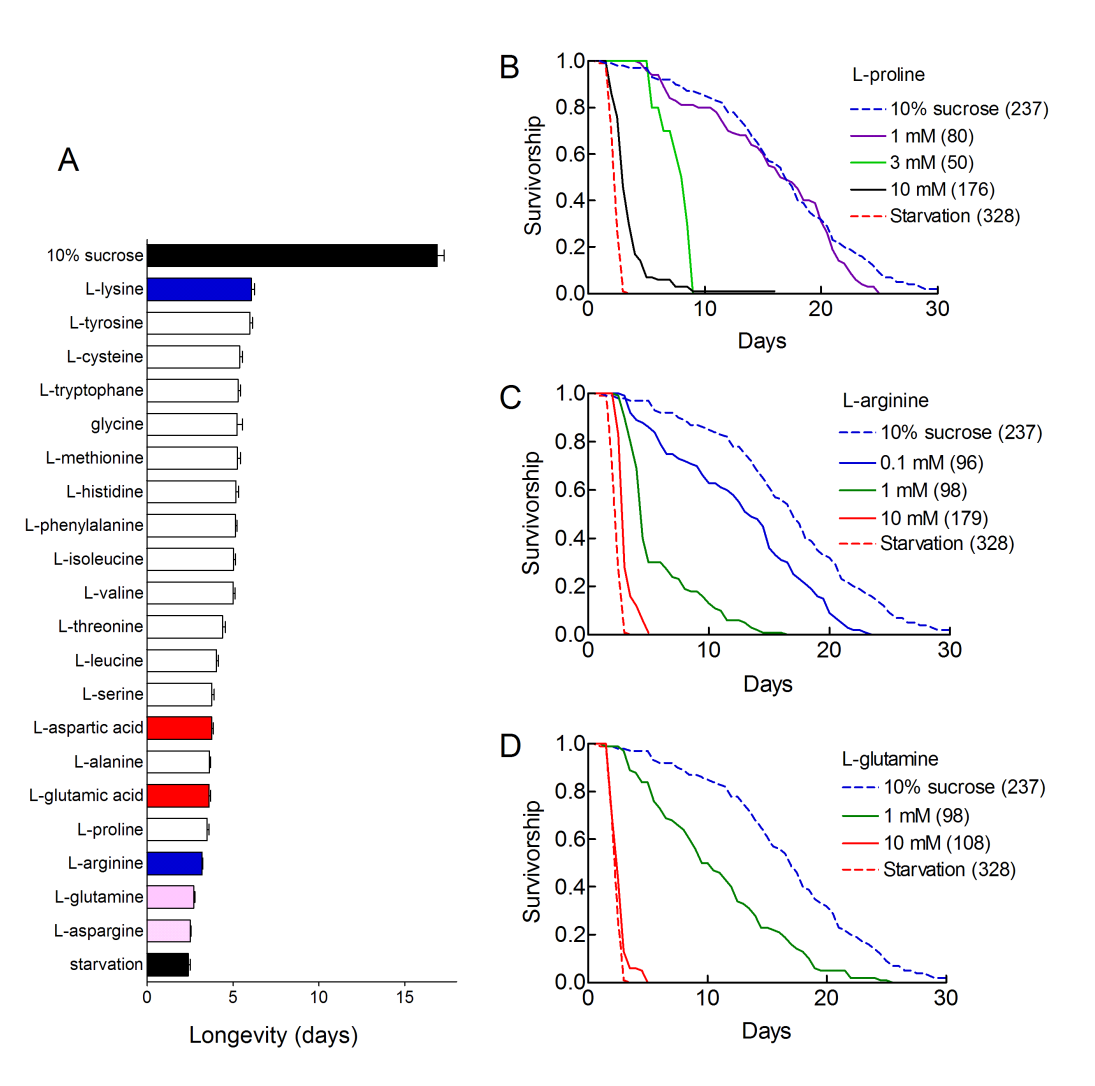
**Natural amino acids reduce the longevity of chronically hypoxic flies**. A. Flies were fed on a 10% sucrose diet supplemented with each individual amino acid (10 mM) and survivorship curves were determined under chronic hypoxic conditions. Mean longevities ± sem are shown. Amino acids are ranked according to their life-shortening effect. The top and bottom black bars show the two control situations which correspond to a pure sucrose diet and starvation conditions. All differences with the pure sucrose condition were statistically significant (p < 0.0001, log rank test). Colour codes were used to label basic amino acids (blue), acidic amino acids (magenta) and amidinated amino acids (red). The total number of flies used was 2341. Sample sizes were 70–237. B-D. Dose dependent actions of L-proline, L-arginine and L-glutamine. Hypoxic flies were fed on a 10% sucrose medium supplemented with the indicated concentrations of amino acids and survivorship curves were determined. The dotted lines show the survivorship curves obtained for hypoxic flies fed on a pure sucrose diet (blue) or under starvation conditions (red). Sample sizes are indicated in parentheses. Note that low mM concentrations of amino acids produced near half maximum reductions in the median and maximum longevities of the flies.

We then asked whether the toxicity of the casein hydrolysate was due to the whole amino acid mixture or to specific amino acid species. We exposed flies to a 10% sucrose diet supplemented with individual amino acids and determined survivorship curves under chronic hypoxic conditions. Amino acids were tested at the same concentration (10 mM). All natural amino acids that compose proteins decreased the median and maximum longevities of hypoxic flies. The most active compounds were L-asparagine and L-glutamine. They reduced the mean survival of the flies to 2.5 – 2.7 days, close to the value observed for starving flies and flies fed on yeast rich or casein rich diets. The least active amino acid was L-lysine. It decreased the mean longevity to 6.1 days. Thus, a complete amino acid mixture was not required to reduce the longevity of hypoxic flies. Any one of the natural amino acids reproduced the action of casein. Figure 2A ranks the different amino acids according to their actions on the mean longevity. This representation shows that no clear relationship could be defined between the life shortening effect of amino acids and their chemical structure.

We then defined the responses of hypoxic flies to different concentrations of L-proline, L-arginine and L-glutamine. The three amino acids reduced the median and maximum longevities of hypoxic flies in dose dependent manners (Figure 2B to 2D). Half maximum actions were observed at low mM concentrations. These potencies can be compared to that of casein. A 0.01% solution of casein hydrolysate, which produced a half maximum decrease in longevity, corresponded to a 1 mM solution of amino acids (considering that the average molecular weight of an amino acid is 100 Da). Thus, L-proline, L-arginine and L-glutamine were as potent as a casein hydrolysate. This indicated that amino acids quantitatively accounted for the action of casein. Yeast was 16 times less potent (Figure 1B). Different reasons may account for the lower potency of yeast. (i) Yeast provided less amino acid on a weight basis than the casein hydrolysate. Indeed yeast extracts are made up of only 50% proteins. (ii) Free amino acids of the casein hydrolysate were more readily accessible to the flies than the undigested proteins provided by yeast powder. (iii) Some yeast component antagonized the toxicity of dietary proteins. For instance, odours from yeast have been shown to modulate the longevity extending effects of dietary restriction [9]. These results clearly caution about the use of poorly defined nutrient mixtures such as yeast extracts in longevity studies. The mechanism by which yeast decreased the longevity of hypoxic flies fed on a sucrose diet was not investigated further. We checked that, as expected, individual amino acids (10 mM) were not toxic to normoxic flies. Clearly, their toxicities required hypoxic conditions to be observed.

### L-citrulline, L-ornithine and polyamines increased hypoxic death

**Figure 3:**
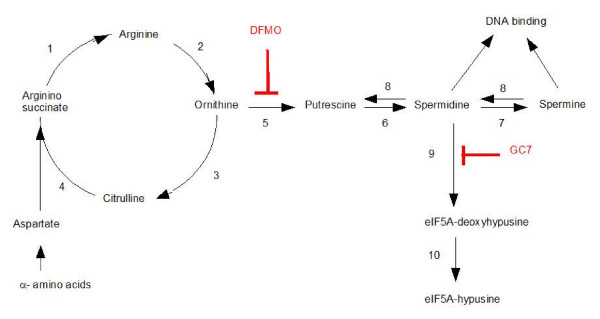
**The synthesis of polyamines and the hypusination of eIF5A**. Enzymes are: arginosuccinate lyase (1), arginase (2), ornithine carbamoyl transferase (3), arginosuccinate synthase (4), ornithine decarboxylase (ODC) (5), spermidine synthase (6), spermine synthase (7). Spermine synthase and spermidine synthase also require decarboxylated S-adenosylmethionine which is produced by S-adenosylmethionine decarboxylase. Polyamines catabolism (8) is achieved by spermine/spermidine N^1^-acetyltransferase and a peroxisomal flavoprotein polyamine oxidase. The formation of hypusine (N^ε^-(4-amino-2-hydroxybutyl)lysine) occurs in two steps. Deoxyhypusine synthase (9) transfers the 4-aminobutyl moiety of spermidine to the ε-amino group of a specific lysine residue of eIF5A. Deoxyhypusine hydroxylase (10) hydroxylates eIF5A-deoxyhypusine. In yeast and mammals, only hypusinated forms of eIF5A are active. DFMO inhibits ODC, the rate limiting enzyme for polyamine synthesis. GC7 inhibits deoxyhypusine synthase (DHS). The polyamines synthetic enzymes, ODC and S-adenosylmethionine decarboxylase are up regulated in response to a decrease in polyamines. Spermine/spermidine N^1^-acetyltransferase which mediates the retroconversion of polyamines is up regulated by spermine and spermidine.

**Figure 4:**
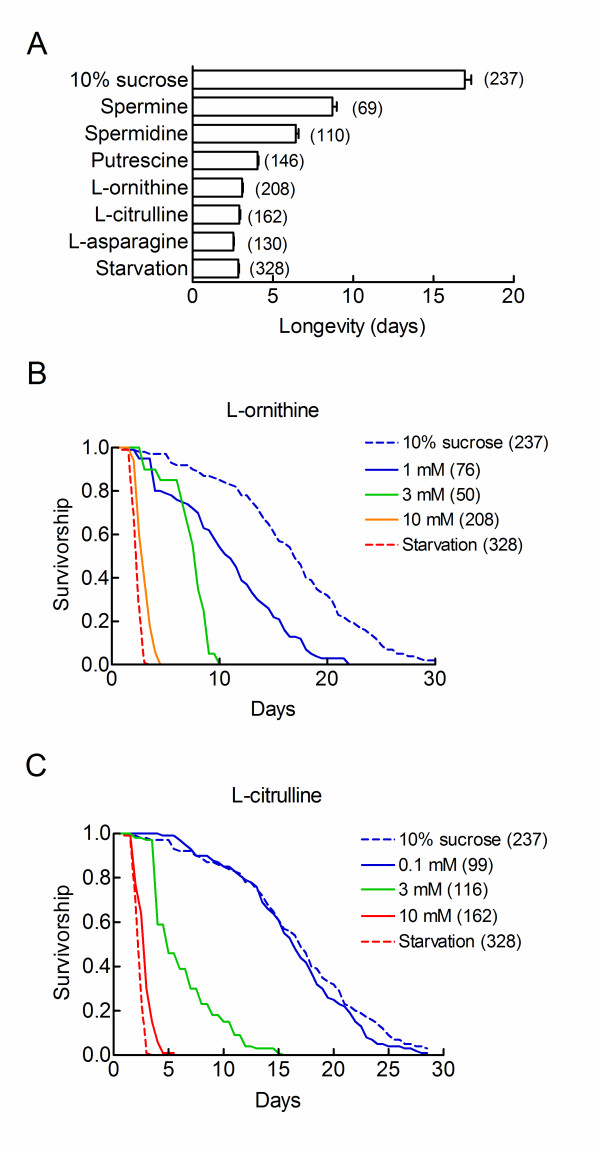
**Life-shortening effects of L-ornithine, L-citrulline and polyamines**. A. Hypoxic flies were fed on a 10% sucrose medium supplemented with 10 mM of the indicated compounds and survivorship curves were determined. Mean longevities ± sem are shown. Sample sizes are indicated in parentheses. B, C: Dose dependent actions of L-ornithine (B) or of L-citrulline (C) on the survival of hypoxic flies. Hypoxic flies were fed on a 10% sucrose diet supplemented with the indicated concentrations of amino acids and survivorship curves were determined. The dotted lines show the survivorship curves obtained for flies fed on pure sucrose diet (blue) or under starvation conditions (red). Sample sizes are indicated in parentheses. Note that low mM concentrations of L-ornithine and L-citrulline produced near half maximum reductions in the median and maximum longevities of the flies. Survivorship curves of flies fed on 10 mM putrescine or 10 mM spermidine are presented in Figure 5 (E and F).

The absence of clear structure activity relationship (Figure 2A) suggested that amino acids might act *via *a very general metabolic pathway. The urea cycle is a likely candidate for the most active amino acids are closely linked to the urea cycle. Arginine is an intermediate of the cycle. Aspartic acid is a substrate for argininosuccinate synthase. It is produced from oxalacetate using transaminating reactions (Figure 3). The urea cycle comprises two amino acid intermediates which are not incorporated into proteins: L-ornithine and L-citrulline. We therefore asked whether L-citrulline and L-ornithine also reduced the longevity of hypoxic flies. Survivorship curves were established for hypoxic flies fed on a 10% sucrose diet supplemented with 10 mM L-citrulline or L-ornithine. Figure 4 shows that the two amino acids decreased the median and maximum longevities of hypoxic flies to the same extent as starvation conditions. Their actions were dose dependent. Half maximum actions were observed at low mM concentrations as observed for natural amino acids (Figure 2). Thus, dietary L-ornithine and L-citrulline reproduced all actions of individual amino acids and casein.

### Pharmacological evidence for a role of polyamines in hypoxic tolerance

One well known function of L-ornithine is to feed the polyamine synthetic pathway (Figure 3). We therefore asked whether polyamines reduced the longevity of hypoxic flies. Figure 4A shows that putrescine, spermine and spermidine decreased the mean longevities of hypoxic flies fed on a pure sucrose diet. Mean longevities were 4–8 days, similar to the values observed in the presence of the least active amino acids. Putrescine, spermine and spermidine were well tolerated by normoxic flies. They did not induce an excess mortality in the normoxic toxicity assay.

**Figure 5:**
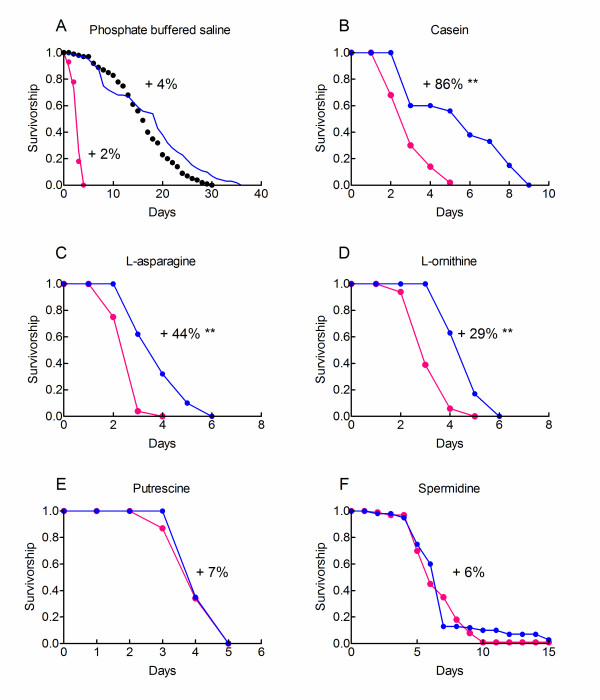
**DFMO prevents actions of amino acid but not of polyamines**. Hypoxic flies were fed on a 10% sucrose diet supplemented with 10 mM of the indicated compounds and survivorship curves were determined. Experiments were performed in the absence or the presence of 10 mM DFMO. Percentage changes in longevity by DFMO are indicated for each dietary condition. A log rank test was used to compare survivorship curves. **: p < 0.0001, no asterisk: p > 0.001. A. Control conditions. Curves on the left are survivorship curves obtained under starvation conditions in the absence (dots, n = 50) or the presence (curve, n = 80) of 10 mM DFMO. Curves on the right are survivorship curves obtained with a 10% sucrose medium in the absence (dots, n = 237) or the presence (curve, n = 118) of 10 mM DFMO. B-F. 10% sucrose diet supplemented with 0.1% casein or 10 mM amino acid or polyamine as indicated. Experiments were performed in the absence (magenta) or the presence of 10 mM DFMO (blue). Sample sizes were B (control, 189; DFMO, 48), C (control, 130; DFMO, 88), D (control, 208; DFMO, 59), E (control, 146; DFMO, 48) and F (control, 110; DFMO, 60).

Ornithine decarboxylase (ODC) is the rate limiting enzyme for polyamine synthesis in mammals. DFMO (α-difluoromethylornithine) is a specific inhibitor of ODC in mammals [10] and insects [11]. Figure 5 shows that DFMO increased the median and maximum longevities of hypoxic flies fed on casein, L-asparagine or L-ornithine. Percentage changes in the mean longevity by DFMO were 29 to 86%. We observed however that flies exposed to DFMO were shorter lived than flies fed on a pure sucrose diet (14.2 days). Thus, DFMO partially reversed the life-shortening effect of casein and amino acids.

Figure 5A shows that DFMO did not modify the longevity of starving hypoxic flies or of hypoxic flies fed on a sucrose diet. Thus, DFMO acted specifically on hypoxic flies that were fed on amino acids.

Figure 5E and 5F further shows that DFMO did not increase the longevity of hypoxic flies fed on sucrose diet supplemented with putrescine or spermidine. These results were not surprising. Putrescine and spermidine are located downstream of ODC in the polyamine synthesis pathway (Figure 3) and their actions should be insensitive to DFMO. Taken together these results suggested that polyamine synthesis probably contributed to the life-shortening effect of dietary amino acids.

### Polyamines and HIF-1α/Sima stabilization

Hypoxia-inducible factor 1 (HIF-1) is a heterodimeric transcription factor that functions as a master regulator of oxygen homeostasis in mammals [12]. The *Drosophila *homologue of HIF-1α is similar (sima). It is now well established that an accumulation of HIF-1α/sima under hypoxic conditions promotes hypoxic tolerance [13]. Recent studies have suggested a possible mechanism that may link polyamines and HIF-1α dependent hypoxic adaptation. Spermidine/spermine acetyl transferase is the rate limiting enzyme for polyamine retroconversion (Figure 3). It is induced by spermidine and spermine [14]. It associates to HIF-1α and reduces its activity by promoting its ubiquitination and its degradation by the proteasone [15,16]. We therefore asked whether HIF-1α/sima contributed to the actions of dietary amino acids and polyamines on chronically hypoxic flies.

HIF-1α/sima signalling in response to chronic hypoxia was evaluated using the endogenous lactate dehydrogenase activity of adult *w*^1118 ^flies. We also used adult LDH-LacZ reporter flies in which expression of β-galactosidase was placed under the control of a strong hypoxia sensitive, murine LDH-A enhancer [17]. We observed that chronic hypoxic conditions (5% O_2_, 24 or 48 hours) did not increase activities of β-galactosidase or of endogenous lactate dehydrogenase. Anoxic conditions (< 0.1% O_2_, 1–2 hours) increased activities of the reporter proteins about 2 fold. These results indicated that hypoxic signalling in adult flies only responded to strong anoxic stresses.

**Figure 6:**
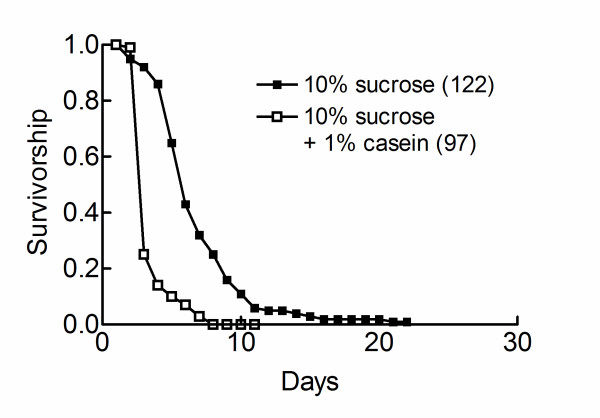
**Casein decreases the longevity of sima loss of function mutants**. Sima^07607^/^07607 ^flies were raised under normoxic conditions. Newly emerged adults were shifted to chronic hypoxic conditions and maintained either on a pure 10% sucrose diet or a 10% sucrose diet supplemented with 1% casein and survivorship curves were determined. Sample sizes are indicated in parentheses. Survivorship curves were compared using the log-rank test. P was < 0.0001. Corresponding survivorship curves for w^1118 ^flies are presented in Figure 1C.

We then used sima loss of function mutants (sima^07607^/^07607^). These flies develop normally under normoxic conditions. They did not under hypoxic conditions, meaning a decreased hypoxic tolerance [13]. Sima^07607^/^07607 ^flies were raised under normoxic conditions and newly emerging adults were exposed to chronic hypoxic conditions. Figure 6 shows that dietary casein decreased the longevity of hypoxic sima^07607^/^07607 ^flies fed on a 10% sucrose diet. Thus, the life-shortening effect of casein did not require an active sima.

### GC7 prevented amino acid and polyamine toxicities

A well known function of polyamine synthesis is to provide spermidine for the hypusination of eukaryotic initiation factor 5A (eIF5A) [18]. Hypusine (N^ε^-(4-amino-2-hydroxybutyl)lysine) is a unique amino acid that occurs only in eiF5A, both in mammals [18] and *Drosophila *[19]. It is formed by a two step mechanism (Figure 3). First, deoxyhypusine synthase (DHS) transfers the 4-aminobutyl moiety of spermidine to the ε-amino group of a specific lysine residue of inactive eIF5A. Deoxyhypusine hydroxylase then hydroxylates eIF5A-deoxyhypusine into eIF5A-hypusine which is the active form of eIF5A. Potent inhibitors of DHS have been developed and are now established tools to assess the functional role of eIF5A hypusination. The most potent inhibitor known so far is GC7, a guanylated derivative of 1,7-diaminoheptane (DAH) [20].

**Figure 7:**
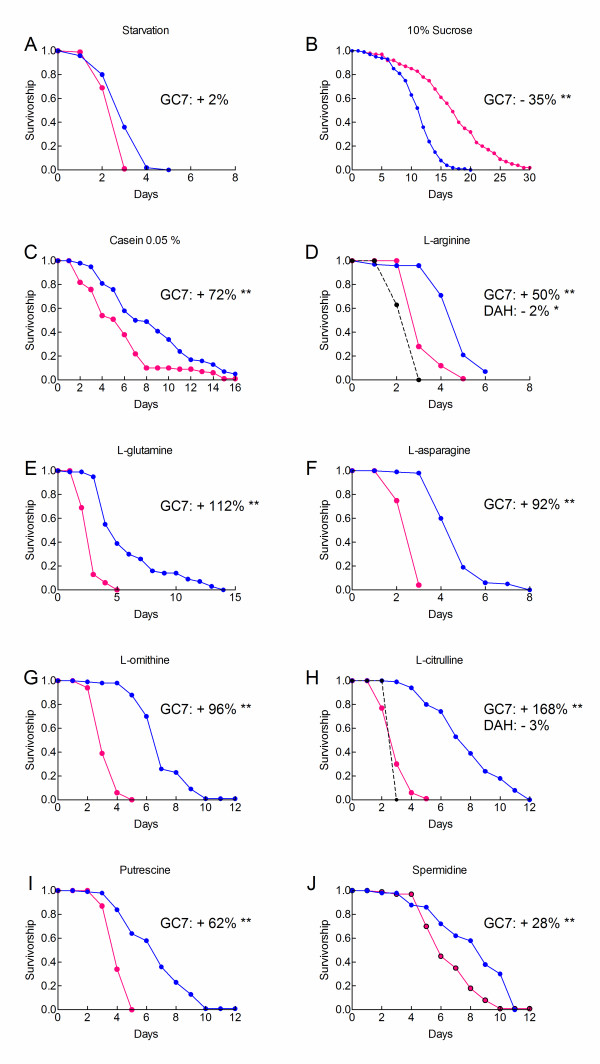
**GC7 prevents actions of casein, amino acids and polyamines**. Survivorship curves were determined in the absence (magenta) or the presence of 10 mM GC7 (blue) or of 10 mM DAH (dotted line in panels D and H). Percentage changes in longevity by GC7 or DAH are indicated for each dietary condition. A log rank test was used to compare survivorship curves. **: p < 0.0001, *: p < 0.001, no asterisk: p > 0.001. Dietary conditions were: A, starvation, B, 10% sucrose diet, C, 10% sucrose diet supplemented with 0.05% casein, D-F, 10% sucrose diet supplemented with 10 mM amino acids or polyamines as indicated. Sample sizes were: A (50 each), B (control, 237; GC7, 99), C (control, 79; GC7, 86), D (control, 179; GC7, 135; DAH, 40), E (control 108; GC7, 103) and F (control, 130; GC7, 193), G (control, 208; GC7, 80), H (control, 208; GC7, 80), I (control, 162; GC7, 89; DAH, 40) and J (control, 110; GC7, 50).

We asked whether GC7 modified the responses of hypoxic flies to amino acids and polyamines. Figure 7A shows that GC7 (10 mM) did not modify the longevity of hypoxic, starving flies. Figure 7B shows that GC7 decreased the longevity of hypoxic flies which were fed on a 10% sucrose diet. Figure 7(C–J) further shows that GC7 increased the median and maximum longevities of hypoxic flies fed on casein, L-arginine, L-glutamine, L-asparagine, L-ornithine, L-citrulline, putrescine and spermidine. Percentage changes in mean longevity by GC7 were 28 to 168%. We observed however that flies exposed to GC7 were shorter lived than hypoxic flies fed on a pure sucrose diet (14.2 days). Thus, GC7 partially reversed the life-shortening effects of casein, amino acids and polyamines.

We then used DAH which is chemically related to GC7 and is a 500 times weaker inhibitor of DHS [20]. DAH did not reduce the life-shortening effects of L-arginine (Figure 7D) or of L-citrulline (Figure 7H). These results suggested that GC7 protected hypoxic flies against amino acid toxicity by a DHS dependent mechanism.

### The hypoxic sensitivity of flies of different strains

**Figure 8:**
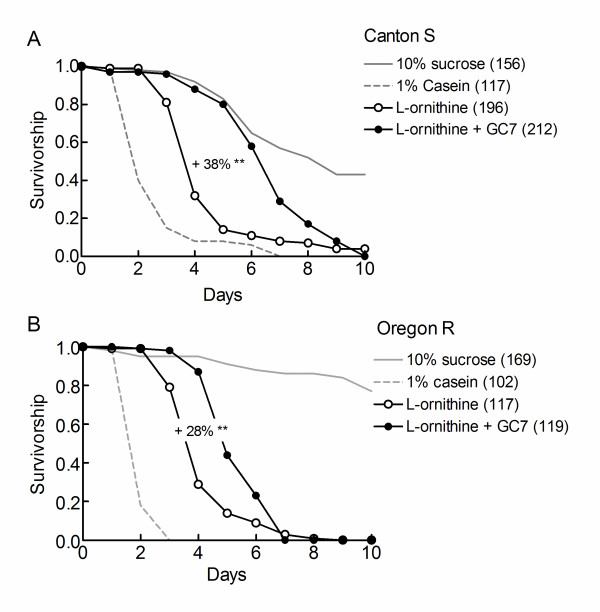
**The longevities of hypoxic Canton S and Oregon R flies are diet and GC7 sensitive**. Flies were fed on different diets as indicated and survivorship curves were defined under chronic hypoxic conditions. Sample sizes are indicated in parentheses. L-ornithine and GC7 were used at 10 mM. Percentage changes in longevity by GC7 are indicated. A log rank test was used to compare survivorship curves: **: p < 0.0001. The survivorship curves of flies fed on a 10% sucrose medium were truncated for clarity. Median and maximum longevities of hypoxic flies fed on a 10% sucrose diet were: Canton S, 8 days and 22 days, Oregon R, 14 days and 22 days.

The responses of *Drosophila *to changes in diet are well known to be highly dependent on genetic background [21]. It was important to determine that the conclusions obtained with the *w*^1118 ^flies were strain independent. Figure 8 shows that the longevities of hypoxic Canton S and Oregon R flies were shortened by casein and by L-ornithine. In addition, GC7 relieved the life-threatening effect of L-ornithine in the two strains of flies.

## Discussion

Chronic hypoxic conditions unmask an unexpected life-shortening effect of dietary proteins and amino acids [6,8]. This paper uses pharmacological tools to analyse the mechanisms involved. First we observed that any one of the amino acids which are incorporated into proteins decreased the longevity of hypoxic flies fed on a sucrose diet. Their half maximum actions are observed at low mM concentrations. The concentrations of amino acids provided by standard food media such as the one used to maintain our stocks of flies are 100 times larger (1.7% yeast, ~80 mM). Thus, low "physiological" concentrations of amino acids such as the one experienced under natural conditions decrease the longevity of hypoxic flies. It is important to stress that a life-shortening effect of dietary amino acids is only observed under chronic hypoxic conditions. Dietary amino acids are required for the normal development of the flies and they increase the longevity of normoxic adult flies [6].

Polyamine metabolism is an evolutionary conserved metabolic pathway. In mammals, polyamines are essential for normal cell growth and differentiation [22,23]. Pharmacological depletion of tissue polyamines and the inhibition of eIF5A hypusination are usually cytostatic and antiapoptotic [24-27]. Polyamine metabolism in insects is less documented. All enzymes of the polyamine synthetic pathway are encoded by the *Drosophila *genome. Spermidine is major form of polyamines in developing *Drosophila *[28] and transcripts for ODC are expressed in tissues from adult flies [29]. Gutfeeling, is the *Drosophila *homolog of antizyme, an endogenous inhibitor of ODC. Mutations in *gutfeeling *cause defects in embryonic peripheral and central nervous systems [30]. Treatment of adult crickets (*Acheta domesticus*) with DFMO decreases spermidine levels and this decrease can be overcome by exogenous putrescine [11]. In the same species, putrescine has a mitogenic effect on mushroom body neuronal precursors. Spermidine and spermine do not induce neuroblast proliferation but they induce neurite outgrowth [31]. Taken together, these studies indicate that polyamines are required for the normal development of insects. We further show here that adult normoxic flies tolerate large amounts of polyamine in their food. This is not surprising for wild flies live on rotten fruits which are full of polyamines. The opposite effect is observed under chronic hypoxic conditions. Putrescine, spermidine and spermine decrease the longevity of chronically hypoxic flies.

The pharmacological evidence presented here suggests that the synthesis of polyamines contribute to the life shortening effect of amino acids.

(i) Actions of yeast extracts, casein and individual amino acids are reproduced by L-ornithine, L-citrulline, two amino acid intermediates of the urea cycle and by polyamines (putrescine, spermine and spermidine). L-ornithine and L-citrulline are as potent as natural amino acids. Polyamines are less potent than amino acids. The difference probably results from pharmacokinetic reasons. Amino acids and polyamines enter cells via different transporters [32]. They may be metabolised to different extents before reaching their targets.

(ii) Inhibition of ODC with DFMO prevents the toxicity of a casein hydrolysate and of all individual amino acids that have been tested. DFMO does not prevent actions of putrescine or of spermidine which are located downstream of ODC in the polyamine synthetic pathway (Figure 3).

Polyamines have several molecular targets and they often act by more than one type of mechanism [22-27]. (i) They interact with nucleic acids and regulate the transcription of a number of genes including the antizyme, an endogenous ODC inhibitor [33] and spermidine/spermine N^1^-acetyltransferase, the rate limiting enzyme for polyamine retroconversion [14]. (ii) Polyamines interact with phospholipids and negatively charged protein domains. They influence the activity of ion channels such as NMDA receptors, AMPA receptors, K^+ ^channels and Ca^2+ ^channels [22]. They activate kinases such as casein kinase and Cdc7 [34]. (iii) Finally, spermidine is a precursor for the hypusination of eIF5A (Figure 3). We show here that the toxicities of amino acids and polyamines are partially prevented by GC7, a specific inhibitor of DHS. A chemically related drug, DAH, is inactive. These suggest that DHS contributes to the life-shortening effect of amino acids, possibly by promoting hypusination of eIF5A. The contribution of other targets of polyamines cannot be excluded.

We also observed DFMO and GC7 are unable to fully prevent the toxicities of amino acids and polyamines. Different reasons may account for their partial actions.

(i) DFMO and GC7 act as competitive antagonists of ODC and DHS respectively [10,20]. As a consequence, their potency *in vivo *can be limited by the presence of large concentrations of enzyme substrates (L-ornithine and spermidine).

(ii) GC7 by itself reduces the survival of sucrose fed hypoxic flies. This effect may limit its beneficial action when flies are fed on amino acids.

(iii) It is well known that polyamine metabolism is highly complex in mammals and that perturbations of polyamine synthesis induce compensatory changes that eventually reduce the efficacy of the drugs used [33,35,36].

(iv) Finally, the possibility that polyamines reduce the longevity of chronically hypoxic flies by more than one mechanism cannot be excluded.

A well known action of polyamines in mammals is to stimulate protein synthesis [37]. A simple hypothesis could be that high rates of protein synthesis are toxic to energetically compromised hypoxic tissues. Accordingly reducing translation could promote survival by sparing energy. This hypothesis is unlikely. 

(i) Single amino acid species produce the same effect as a casein hydrolysate; they are unable to support protein synthesis. 

(ii) Inhibiting translation with cycloheximide does not reproduce the actions of DFMO or of GC7 (data not shown). 

(iii) Although eIF5A has initially been described as a general translation factor [38], more recent evidence suggests that hypusination of eIF5A does not regulate the overall rates of protein synthesis both in *Drosophila *[39] and mammalian cells [40]. Other functions of eIF5A have been proposed. They are nuclear export and RNA turnover [41,42], p53 dependent apoptosis [43] and neuronal survival [44]. Further work is required to define the possible contributions of these different mechanisms to amino acid dependent hypoxic tolerance.

Results presented here identify a link between the synthesis of polyamines and the longevity of chronically hypoxic *Drosophila*. Circumstantial evidence suggests that a similar link probably exist in mammals. For instance it has been reported that activity of ODC and tissue polyamines levels increase in different models of cardiac and brain ischemia [45-47]. In addition, DFMO has neuroprotective [46,48] and cardioprotective [49] actions in mammals. The molecular mechanisms involved have not been analyzed. The possibility that inhibition of polyamine synthesis protects ischemic mammalian tissues by preventing eIF5A hypusination has not yet been considered.

## Conclusion

Chronic hypoxic conditions unmask a life-shortening effect of dietary amino acids. Pharmacological evidence obtained so far suggests that the synthesis of polyamines and the hypusination of eIF5A contribute to the life-shortening effect of dietary amino acids. The results also indicate that drugs can be designed to specifically enhance the hypoxic tolerance of an organism.

## Methods

### Fly Strains

*w*^1118 ^flies were used in all experiments unless otherwise indicated. This strain is long lived under normoxic conditions. The mean longevity of w^1118 ^flies maintained under optimum diet conditions (10% sucrose and 5% yeast) was 78.6 ± 2.2 days (n = 88) [6]. Oregon R and Canton S flies were used in some experiments. All flies were obtained from the Bloomington stock center. Sima^07607^/TM3 flies and LDH-LacZ reporter flies were kindly provided by Dr P. Wappner. Homozygous, loss of function sima ^07607^/^07607 ^flies, were generated by crossing heterozygous sima^07607^/TM3 flies. Sima^07607^/TM3 flies were identified to their stubble phenotype.

All flies were reared in 300 ml bottles filled with 30 ml of standard food medium (8.2% cornmeal, 6.2% sucrose, 1.7% heat inactivated baker's yeast and 1% agar supplemented with 3.75 g/l methyl 4-hydroxybenzoate). They were maintained in humidified, temperature controlled chambers at 25°C, 60% relative humidity and under a 12:12 light: dark cycle.

### Chemicals

N^1^-Guanyl-1,7-diaminoheptane (GC7) was from Research Technologies Inc. (Novato, Ca). All other chemical were purchased from the Sigma Chemical Co (St Louis, Mo).

Survival under chronic hypoxic conditions

All experiments were performed using males. Newly emerging males were collected over a 24 h period and divided into identical batches of 10 males per vial. Vials were sealed with cotton plugs (normoxic conditions) or with natural rubber septa (SubA seal, Sigma, St Louis, Mo) for hypoxic/anoxic conditions. The atmosphere was changed to a 5% O_2 _(hypoxia) or a 0.1% O_2 _(anoxia) atmosphere by flushing vials with premixed gas O_2_/N_2 _mixtures (Linde Gas) [8]. Dead flies were counted every day. Flies were maintained at 25°C and a 12:12 light: dark cycle.

### Dietary manipulations

Solutions of sucrose, heat inactivated yeast, casein hydrolysate (referred to as casein), amino acids, polyamines and inhibitors were prepared at room temperature in phosphate buffered saline. All solutions were prepared immediately before use and were pH checked (7.5). Two ml aliquots were used to wet pieces of Kleenex^® ^towels (1/4 of the original size) that had previously been inserted into the tubes. Nutrient media were labelled according to their sucrose and yeast contents. A 10S10Y nutrient medium contained 10% sucrose (S) and 10% yeast (Y). In starvation experiments (0S0Y nutrient medium), flies were exposed to pieces of Kleenex^® ^towels wetted with 2 ml of phosphate buffered saline to prevent dehydration.

### Toxicity assay

Solutions of drugs (10 mM) in phosphate buffered saline supplemented with 10% sucrose were prepared and used to wet pieces of Kleenex^® ^tissue. Ten, one day old, male flies were added to each tube and tubes were sealed with cotton plugs. Dead flies were counted after 10 days. The 10 day mortality rate was 2% in the absence of drugs. It was < 5% in the presence of amino acids, polyamines or drugs.

### Beta-galactosidase assay

Frozen LDH-LacZ reporter flies were homogenized into lysis buffer (50 mM Hepes, 5 mM CHAPS at pH 7.5) at 4°C. Extracts were centrifuged for 30 minutes at 13,200 rpm. The supernatant was recovered. Beta-galactosidase activity was measured on 100 μg of protein using the beta-galactosidase reporter gene activity kit (Sigma Chemical Co. St Louis, Mo). The time of incubation at 37°C was 1 hour.

### Lactate dehydrogenase activity

Frozen *w*^1118 ^flies were homogenized in phosphate buffered saline (pH 7.5) supplemented with 0.12 M mannitol, 0.22 M sucrose and 1 mM EDTA at 4°C. Extracts were centrifuged for 30 minutes at 13,200 rpm. The supernatant was recovered. Lactate dehydrogenase activity was measured spectrophotometrically at 25°C and using 200 μg of protein. The incubation medium was phosphate buffered saline (pH 7.5) supplemented with 30 μM pyruvate and 15 μM NADH.

### Protein assays

Proteins were measured using the Bradford reagent (Biorad Res. Labs, Hercules, Ca).

### Statistical analysis

Complete survivorship curves were defined for all conditions considered. Survivorship curves were compared using the logrank test and the GraphPad Prism 4 software (San Diego, Ca). For each condition we calculated the mean, median and maximum longevities. The maximum longevity was defined as the median longevity of the final surviving 10%. Dose response curves for the actions of yeast and casein on the mean survival of hypoxic flies were fitted to sigmoidal curves using the GraphPad Prism 4 software.

## Authors' contributions

PV and CF designed and performed the experiments. CF wrote the paper.
